# Transglutaminase 2 crosslinks the glutathione S-transferase tag, impeding protein–protein interactions of the fused protein

**DOI:** 10.1038/s12276-020-00549-9

**Published:** 2021-01-13

**Authors:** Hyo-Jun Kim, Jin-Haeng Lee, Ki Baek Lee, Ji-Woong Shin, Mee-ae Kwon, Soojin Lee, Eui Man Jeong, Sung-Yup Cho, In-Gyu Kim

**Affiliations:** 1 Department of Biochemistry and Molecular Biology, Seoul National University College of Medicine, Seoul, Korea; 2 Department of Pharmacy, College of Pharmacy, Jeju National University, Jeju Special Self-Governing Province, Korea; 3 Department of Biomedical Sciences, Seoul National University College of Medicine, Seoul, Korea; 4 Institute of Human-Environment Interface Biology, Seoul National University College of Medicine, Seoul, Korea

**Keywords:** Protein purification, Expression systems

## Abstract

Glutathione S-transferase (GST) from *Schistosoma japonicum* has been widely used as a tag for affinity purification and pulldown of fusion proteins to detect protein–protein interactions. However, the reliability of this technique is undermined by the formation of GST-fused protein aggregates after incubation with cell lysates. It remains unknown why this aggregation occurs. Here, we demonstrate that the GST tag is a substrate of transglutaminase 2 (TG2), which is a calcium-dependent enzyme that polyaminates or crosslinks substrate proteins. Mutation analysis identified four glutamine residues in the GST tag as polyamination sites. TG2-mediated modification of the GST tag caused aggregate formation but did not affect its glutathione binding affinity. When incubated with cell lysates, GST tag aggregation was dependent on cellular TG2 expression levels. A GST mutant in which four glutamine residues were replaced with asparagine (GST^4QN^) exhibited a glutathione binding affinity similar to that of wild-type GST and could be purified by glutathione affinity chromatography. Moreover, the use of GST^4QN^ as a tag reduced fused p53 aggregation and enhanced the induction of p21 transcription and apoptosis in cells treated with 5-fluorouracil (5-FU). These results indicated that TG2 interferes with the protein–protein interactions of GST-fused proteins by crosslinking the GST tag; therefore, a GST^4QN^ tag could improve the reproducibility and reliability of GST pulldown experiments.

## Mycobacteria: Regulatory and signalling molecules that promote inflammation

A small RNA molecule which controls the expression of genes, and cell signaling molecules called cytokines are associated with the damaging inflammation caused by mycobacteria. Mycobacterial infections, which can affect most organs of the body, and are often resistant to most available drugs, are emerging as a serious global health concern. Patients with suppressed immunity are at particular risk. Increased production of a specific microRNA and of a variety of cytokines during mycobacterial infection has been demonstrated by Hyeon Ji Kim and colleagues at Chungnam National University, Daejon, and co-workers from other institutions in South Korea. Their findings come from comparing peripheral blood mononuclear cells in patients with *Mycobacteroides abscessus* lung infections with those from healthy controls. The researchers found similar increased microRNA and cytokine production in the lungs of infected mice.

## Introduction

Glutathione-S-transferase (GST) from *Schistosoma japonicum* is often included as a protein tag in a commonly used molecular biology technique. The GST tag enhances the solubility of fusion proteins expressed in *Escherichia coli* and is used to purify fusion proteins due to its ability to bind to immobilized glutathione (GSH) on agarose beads^1^. Historically, GST fusion proteins purified by GSH affinity chromatography have been used to identify new protein–protein interactions. In this case, cell lysates are incubated with a GST fusion protein, and subsequent GST pulldown experiments are performed^2^. However, high molecular-weight aggregates are often detected by the anti-GST antibody after protein separation by SDS-PAGE. The formation of aggregates is variable and inconsistent and depends on the cell type used and on the experimental conditions of the GST pulldown assay. GST fusion protein aggregates interfere with reproducible protein–protein interactions and thus undermine the reliability of the GST pulldown experiment. Notably, the mechanism for the aggregate formation of the GST fusion protein remains poorly understood.

Transglutaminase 2 (TG2) is a ubiquitously expressed enzyme that catalyzes the calcium-dependent transamidation reaction between glutamine and lysine residues of proteins to form crosslinked proteins. TG2 also catalyzes the incorporation of polyamines, including putrescine, spermidine, and spermine, into the glutamine residues of proteins to produce polyaminated proteins^3^. Previously, we showed that TG2 mediates crystallin, caspase 3, and collagen crosslinking in response to oxidative stress, hypoxic stress, and DNA damage, which leads to cataract formation, apoptotic resistance, and fibrotic development, respectively^4–6^. Moreover, TG2 mediates HPV-E7 and eIF4E-BP polyamination, which affects their respective interactions with Rb and Raptor to suppress HPV-E7 and selectively control translation^7,8^. In addition, TG2 expression is upregulated in many cancer cells^9–12^. During pulldown experiments to identify TG2-interacting proteins and to analyze domain–domain interactions, lysates from cells that express a high level of TG2 exhibited an increase in aggregate formation. Thus, we investigated the role of TG2 in GST fusion protein aggregation.

Here, we demonstrated that the GST tag is a substrate for TG2 and that crosslinking between the GST tag and other TG2 protein substrates is responsible for the formation of GST fusion protein aggregates during pulldown experiments.

## Materials and methods

### Cell culture and transient transfection

HeLa, 293FT, DU145, and H1299 cells were cultured in Dulbecco’s modified Eagle’s medium (DMEM, WelGENE) supplemented with 10% fetal bovine serum (FBS, HyClone) and 1% penicillin-streptomycin (Gibco). MCF7, A549, and HT-1080 cells were cultured in RPMI 1640 medium from WelGENE supplemented with 10% FBS and 1% penicillin-streptomycin. The cells were grown at 37 °C in a humidified incubator containing 5% CO_2_. For transfection, the cells were cultured until 70% confluency and transfected with the pCMV-2B FLAG-GST expression vector using Lipofectamine 3000 reagent in Opti-MEM (Gibco) medium according to the manufacturer’s protocol (Invitrogen).

### 5-(Biotinamido)pentylamine (BP) incorporation assay

Purified GST tag and human TG2 were incubated with BP (Pierce), 5 mM 1,4-dithiothreitol (DTT) (Sigma-Aldrich), and CaCl_2_ (Sigma-Aldrich). In vitro BP incorporation was performed at 37 °C for 1 h, and then, the samples were diluted with sample loading buffer (60 mM Tris-Cl (pH 6.8), 10% glycerol, 2% SDS, 1% β-mercaptoethanol (ME), 0.01% bromophenol blue) and boiled at 100 °C for 10 min. The samples were separated by sodium dodecyl sulfate-polyacrylamide gel electrophoresis (SDS-PAGE) and transferred to nitrocellulose (NC) membranes (GE Healthcare). After the membrane was blocked with 5% skim milk TBST (20 mM Tris-Cl (pH 7.4), 150 mM NaCl, 0.1% Tween-20), it was incubated with streptavidin (SA) coupled to horseradish peroxidase (HRP) (1:1000, Pierce) and washed three times with TBST for 10 min. The membrane was visualized by incubation with Supersignal West Pico solution (Pierce) and exposure to X-ray film (Agfa).

### Well plate TG activity assay

The in situ TG activity was measured based on published protocol^13^ with modifications. Briefly, cells were incubated with 500 μM BP for 1 h. Then, prepared cell lysates were plated on a 96-well plate (Nunc) overnight at 4 °C and incubated with 5% BSA PBST (NaCl 137 mM, KCl 2.7 mM, Na_2_HPO_4_ 10 mM, KH_2_PO_4_ 1.8 mM, 0.1% Tween 20) for 2 h at room temperature (RT). Streptavidin-HRP containing 1% BSA PBST (1:100) was replaced and incubated for 2 h at RT. The level of BP incorporated into cells was used as an indicator of TG2 activity. After o-phenylenediamine dihydrochloride (OPD, Sigma-Aldrich) addition, the reaction was stopped by supplementation with 1 N H_2_SO_4_. Absorbance was detected by using a microplate spectrophotometer (Bio-Rad) at 490 nm.

### Western blot analysis and Coomassie Brilliant Blue (CBB) staining

Samples were boiled in sample loading buffer at 100 °C for 10 min, separated by SDS-PAGE, and transferred onto NC membranes for blotting. Membrane blocking was performed for 1 h with 5% skim milk in TBST at RT. After blocking, the membranes were incubated with mouse anti-GST (Santa Cruz Biotechnology), TG2^7^, and FLAG antibodies (Sigma-Aldrich) at 4 °C overnight. The membranes were washed with TBST three times each for 10 min. Primary antibodies were detected with anti-rabbit HRP-conjugated secondary antibody (1:1000, Jackson Laboratory) or anti-mouse HRP-conjugated secondary antibody (1:1000, Pierce). The samples were incubated for 1 h at RT, washed three times with TBST, reacted with Supersignal West Pico solution (Pierce) for 5 min, and exposed to X-ray film (Agfa). For CBB staining, SDS-PAGE gels were stained for 10 min using staining solution (0.1% Coomassie Brilliant Blue G250, 10% acetic acid, 50% methanol, and 40% H_2_O_2_) and destained with distilled water overnight.

### Pulldown assay

Wild-type (WT) and mutant GSTs were expressed in Rosetta (DE3) competent cells by 1 mM isopropyl β-D-1-thiogalactopyranoside (IPTG) (Amnesco). For the measurement of GSH binding affinity, samples were applied to GSH-Sepharose beads (GE Healthcare) and incubated for 30 min at RT by rotating. After incubation, the beads were washed with 1 ml of ice-cold phosphate-buffered saline (PBS), pH 7.4, three times, and the beads were eluted in sample loading buffer by boiling for 10 min at 100 °C.

### GST purification

GST proteins are produced from 1 l of *E. coli* Rosetta (DE3) competent cells grown in LB medium. A standard expression induction protocol was based on shifting log-phase cultures (A600 = ~0.6) from 37 °C and adding IPTG (1 mM). After 1 h with constant vigorous shaking, cells were pulled down by centrifugation at 5000 rpm at 4 °C for 10 min. Pelleted bacterial cells were lysed in 30 ml of lysis buffer (20 mM Tris-Cl (pH 7.5), 0.1% Triton X-100, 1 mg/ml lysozyme) for 20 min and sonicated, and insoluble molecules were removed. Then, we placed the soluble lysates into a tumbler at 4 °C with GSH-Sepharose 4B or Ni-NTA resin (GE Healthcare). GSH or Ni-NTA bead washing was performed four times with cold PBS or washing buffer (20 mM Tris-Cl (pH 8.0), 300 mM NaCl, 20 mM imidazole). GST protein elution was conducted with 10 mM reduced GSH in 50 mM Tris pH 8.0 or 250 mM imidazole in 20 mM Tris pH 8.0 (300 mM NaCl). Finally, eluted GST protein dialysis was performed overnight against TBS at 4 °C.

### CDNB enzymatic assay

GST activity was measured using 1-chloro-2,4-dinitro-benzene (CDNB, Sigma-Aldrich) as the substrate. Ten-microliter samples were added to each well of a 96-well plate (SPL), and a 150 μl mixture containing 100 mM KH_2_PO_4_ pH 6.5, 1 mM CDNB, and 1 mM GSH was added to each well. Then, the mixtures were placed onto a microplate spectrophotometer (Bio-Rad) for measurement of the absorbance at 340 nm.

### Site-directed mutagenesis

Site-directed mutagenesis of the GST clone was performed using a QuikChange mutagenesis kit (Stratagene) according to the manufacturer’s protocol. Mutated GST sequences were subcloned into pET-15b, pET-24d, pGEX 4T-1, and pCMV-2B expression vectors, and the mutated constructs were confirmed by DNA sequencing.

### RNA extraction, RT-PCR, and real-time PCR

Total RNA extraction was performed using the Maxime RT Premix Kit (iNtRON Biotechnology) according to the manufacturer’s instructions. Primers were designed with Primer 3 (http://bioinfo.ut.ee/primer3-0.4.0/). Real-time PCR primer pairs were as follows: human p21 (5′-TGTCCGTCAGAACCCATGC-3′ and 5′-AAAGTCGAAGTTCCATCGCTC-3′); human actin (5′-CATGTACGTTGCTATCCAGGC-3′ and 5′-CTCCTTAATGTCACGCACGAT-3′). The cDNA samples were diluted in 30 ng/well. Each PCR was performed in a 20 μl reaction mixture containing 2X KAPA SYBR FAST qPCR Master Mix (KAPA Biosystems). Real-time PCR was performed using a CFX96 Real-Time System (Bio-Rad). The relative gene expression was determined using the 2^−△△Ct^ method^14^.

### WST-1 assay

The WST-1 assay was performed using an EZ-CYTOX assay kit (DAEILLAB SERVICE) according to the manufacturer’s protocol. Briefly, cells (10,000/well) were seeded in 96-well plates. After 5-FU treatment, PBS washing was performed once, and 100 μl of fresh RPMI 1640 medium was added. Ten microliters of WST-1 solution was added to the culture media and incubated for 3 h. Absorbance at 450 nm was measured by a microplate spectrophotometer (Bio-Rad).

### Statistical analysis

Statistical significance was analyzed using the Student’s *t*-test with Prism software (GraphPad). Statistical significance is represented in the figures by *p < 0.05; **p < 0.01; ***p < 0.001.

## Results

### The GST tag is a TG2 substrate

**Fig. 1:**
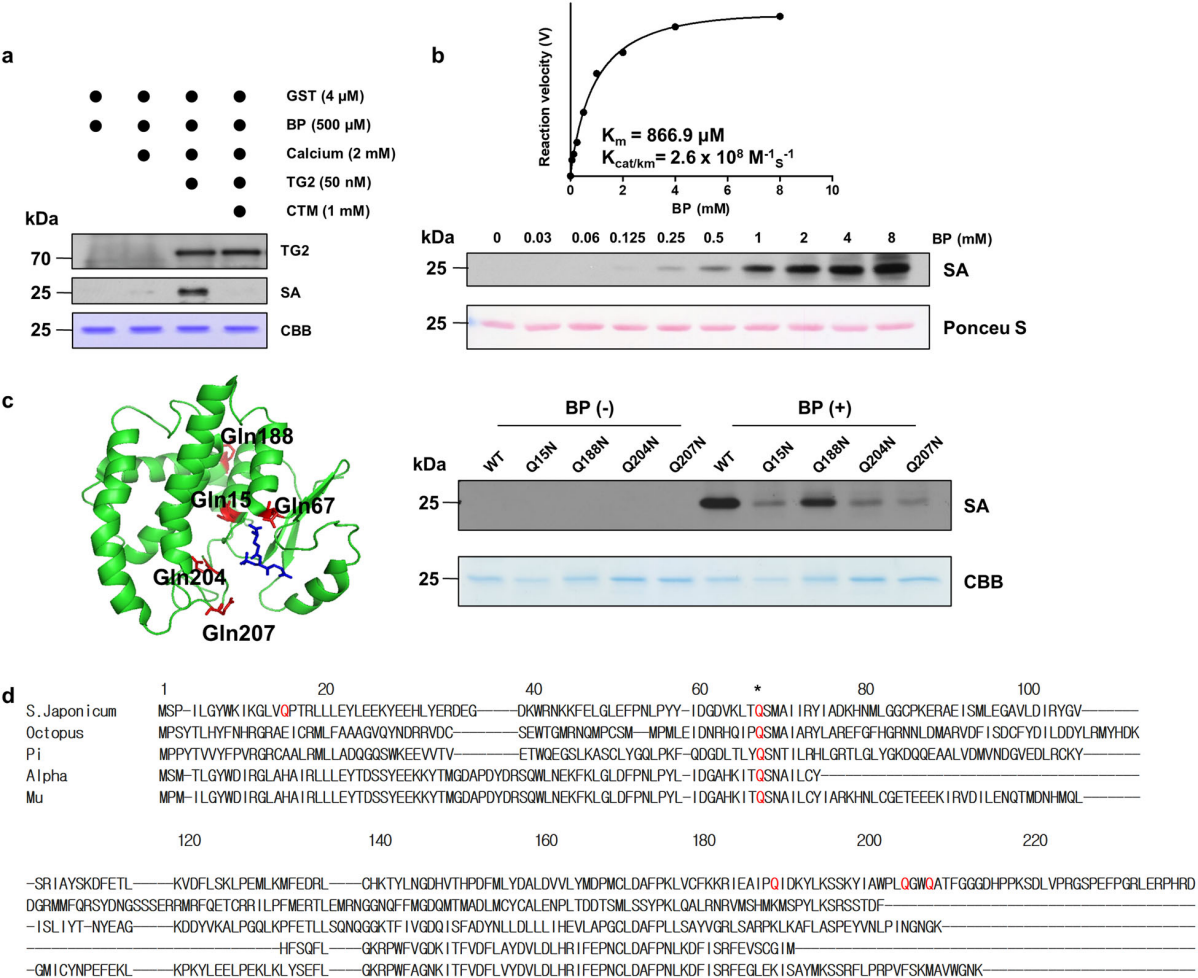
The GST tag is a TG2 substrate. **a** TG2-mediated biotinylated pentylamine (BP) incorporation assay. The purified GST tag (4 μM) was incubated with human TG2 (50 nM), 500 µM BP, and 2 mM CaCl_2_ at 37 °C for 1 h. The reaction mixtures were analyzed by Coomassie Brilliant Blue (CBB) staining and western blotting with streptavidin (SA)-HRP or an anti-TG2 antibody. Cystamine (1 mM, CTM) was used as a TG2 inhibitor. **b** Determination of the GST *K*_m_ value for TG2. The BP incorporation assay was performed with the GST tag (4 μM) in the presence of various BP concentrations (0–8 mM). Immunoblot band intensities were quantified to estimate enzyme velocity. **c** Identification of polyamination sites in the GST tag. Glutamine residues are shown as red sticks, and glutathione is shown as a blue stick in the GST tag crystal structure (PDB: 1M99, left). Wild-type and glutamine-to-asparagine mutants of the GST tag were incubated with TG2 in the presence or absence of BP at 37 °C for 1 h. The BP-incorporated GST tag was then detected by western blot analysis (right). **d** Sequence alignment of GST isoenzymes. Polyamination sites are not conserved in the GST family. The asterisk indicates the conserved glutamine residue (Q^67^).

To determine whether TG2 is involved in the aggregate formation of the GST fusion protein, we investigated whether the GST tag was a TG2 substrate using an incorporation assay that utilized the synthetic polyamine biotinylated pentylamine (BP). The purified GST tag was incubated with recombinant human TG2 and BP in the presence of calcium. Protein in the reaction mixture was then separated by SDS-PAGE, and BP incorporation was detected with streptavidin-HRP (SA-HRP). Western blot analysis revealed that BP was incorporated into the GST protein. Incubation with cystamine (CTM), which is a TG2 inhibitor, completely blocked BP incorporation (Fig. 1a). These data indicated that the GST tag is a TG2 substrate. To determine whether the polyamine levels found in cell lysates are sufficient to incorporate into the GST tag, we determined the *K*_m_ and catalytic efficiency of TG2. Purified GST and TG2 were incubated with various concentrations of BP, and BP incorporation into the GST tag was determined by western blots. The calculated *K*_m_ and *K*_cat_/*K*_m_ values were 866.9 μM and 2.6 × 10^8^ M^−1 ^s^−1^, respectively (Fig. 1b). Because polyamines are found at millimolar concentrations in cells^15^, these results suggest that the GST tag is readily polyaminated or crosslinked by TG2.

In TG2-catalyzed BP incorporation, the glutamine residues on the GST tag act as amine acceptors. Five glutamine residues (Q15, 67, 188, 204, and 207) are present in the GST tag composed of *S. japonicum* GST, which belongs to the Mu class. To identify the polyamination sites on the GST tag, we mutated each of the five glutamine residues to an asparagine (N), and BP incorporation levels of each GST tag mutant were assessed by western blots. Under the same experimental conditions, all mutants except Q67N exhibited reduced BP incorporation. The mutants are herein listed in order of reduced BP incorporation: Q207N, Q204N, Q15N, and Q188N (Fig. 1c and Supplementary Fig. S1), which indicates that Q207 and Q204 are major polyamination sites. Interestingly, the Q67N mutant was not purified with GSH-Sepharose beads. Moreover, sequence alignment showed that only Q67, and not the other polyamination sites, was conserved in GST Alpha, Mu, and Pi, octopus, and *S. japonicum* (Fig. 1d). These results suggest that Q67 may be a critical residue for GSH binding and that TG2-mediated polyamination is a unique feature of the GST tag.

### TG2 induces polymer formation of the GST tag

**Fig. 2:**
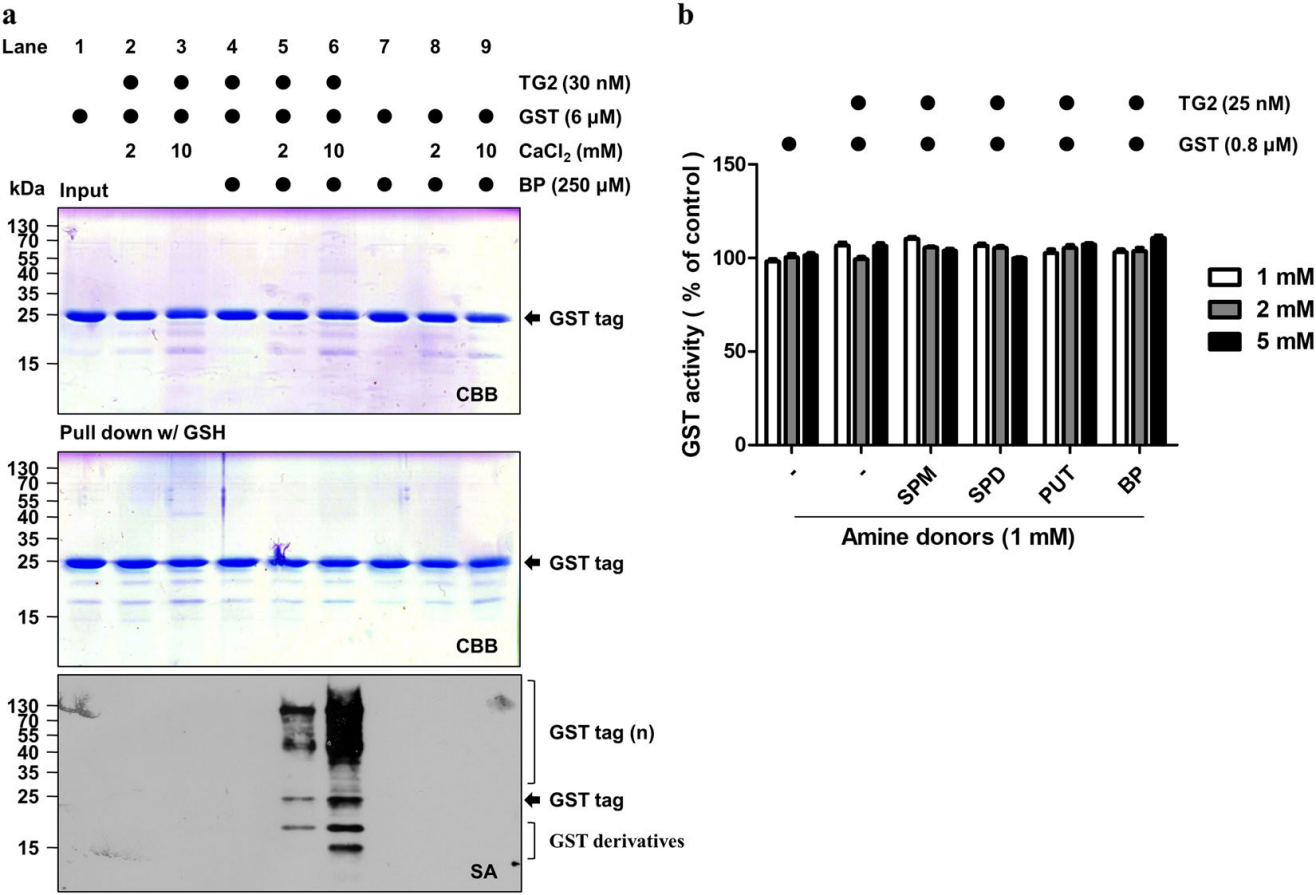
TG2 induces GST tag polymer formation but does not affect its enzymatic activity. **a** TG2-mediated GST tag crosslinking. The purified GST tag (6 μM) was incubated with human TG2 (30 nM), CaCl_2_ (2 or 10 mM), and BP (250 μM) at 37 °C for 1 h. The reaction mixtures were then pulled down with glutathione (GSH)-Sepharose beads. Beads that were bound to the GST tag were quantitated by CBB staining or western blot analysis with SA-HRP. **b** GST activity of the polyaminated GST tag. GST enzymatic activity was measured after reacting TG2 and polyamines using 1-chloro-2,4-dinitro-benzene (CDNB) as a substrate at various CaCl_2_ concentrations. SPM spermine, SPD spermidine, PUT putrescine, BP biotinylated pentylamine. The data are presented as the mean value ± SEM of triplicate experiments.

We next tested whether TG2-mediated polyamination of the GST tag affected the purification efficiency of GST. To this end, GST proteins were incubated with TG2 and BP in the presence of calcium, followed by purification with GSH-Sepharose beads. CBB staining showed that there was no apparent difference in the amount of purified GST protein when a TG2-treated GST tag and control GST tag were compared. However, western blot analysis with SA-HRP revealed that the GST tag was polyaminated and polymerized by TG2. The level of GST tag modification increased with increasing calcium concentrations (Fig. 2a). These data demonstrated that polyaminated or polymerized GST tags can be purified by GSH-Sepharose beads. The data also suggested that TG2-mediated modification does not affect GSH binding affinity to the GST tag. Because GST activity depends on GSH binding, we measured GST enzymatic activity. The GST tag was incubated with various types of polyamines, and GST activity was determined with 1-chloro-2,4-dinitrobenzene (CDNB). Polyamination did not change GST activity (Fig. 2b), which confirmed that a polyaminated GST tag exhibits similar GSH binding affinity.

We also investigated the role of cysteine (Cys) residues of the GST tag in TG2-mediated aggregation because Cys residues are sensitive to cellular redox status. We mutated all four Cys residues in the GST tag to serine (4CS mutant). The 4CS mutant was purified using GSH beads and showed little difference in TG2-mediated polymerization (Supplementary Fig. S2). Therefore, the redox status of Cys residues in the GST tag is not associated with TG2-mediated polymerization.

**Fig. 3:**
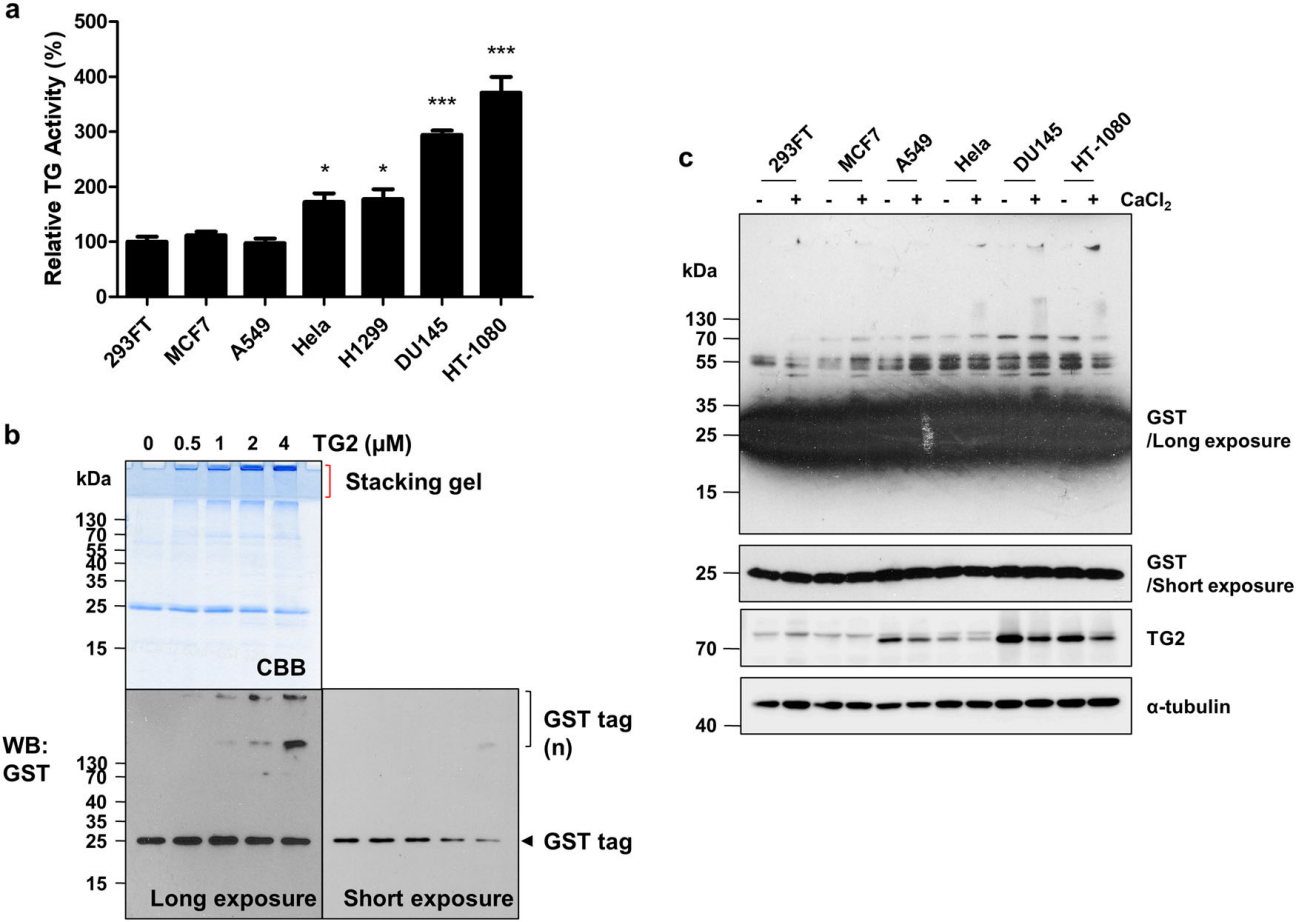
GST tag aggregation depends on TG2 levels in cell lysates. **a** TG2 activity in various cell lines. Cells were cultured in media that contained 500 μM BP for 1 h prior to the assay. TG2 activity was estimated by BP incorporation and was probed with SA-HRP in a well plate assay. **b** TG2-dependent GST tag aggregation. Purified GST tag (4 μM) was incubated with increasing doses of TG2 in the presence of CaCl_2_ (2 mM). Protein in the reaction mixture was separated by SDS-PAGE and analyzed by CBB staining or western blotting with an anti-GST antibody. **c** GST tag crosslinking with cellular proteins. Purified GST tags (5 μg) were incubated with various cell lysates in the presence of CaCl_2_ (10 mM) at 37 °C for 1 h. Protein in the samples was separated by SDS-PAGE and analyzed by western blotting with anti-GST, TG2, and α-tubulin antibodies. Statistical significance is represented in figures by *p < 0.05; **p < 0.01; ***p < 0.001.

We next tested the effect of TG2-mediated polyamination of the GST tag on the reliability of the GST pulldown assay. In cancer cells, TG2 activity (Fig. 3a) and expression (Fig. 3c) were cell line-dependent. HT-1080 cells exhibited the highest TG2 activity among the cell lines that were investigated. We assessed GST tag aggregation levels by increasing purified TG2 amounts. CBB staining and western blot analysis revealed GST tag protein aggregates located on top of the stacking and separating gels. The aggregate quantity appeared to proportionally increase with a reduction in the GST tag monomer (Fig. 3b). These results suggest that the aggregate formation of the GST tag depends on cell line-specific TG2 expression levels. To confirm these results, we incubated GST proteins with lysates from various cell lines. Consistently, the number of GST tag aggregates was highest after incubation with HT-1080 cell lysate (Fig. 3c). Together, these results indicated that TG2 is responsible for the aggregate formation of the GST tag during cell lysate incubation.

### Polyamination-defective GST can be purified by GSH affinity chromatography

**Fig. 4:**
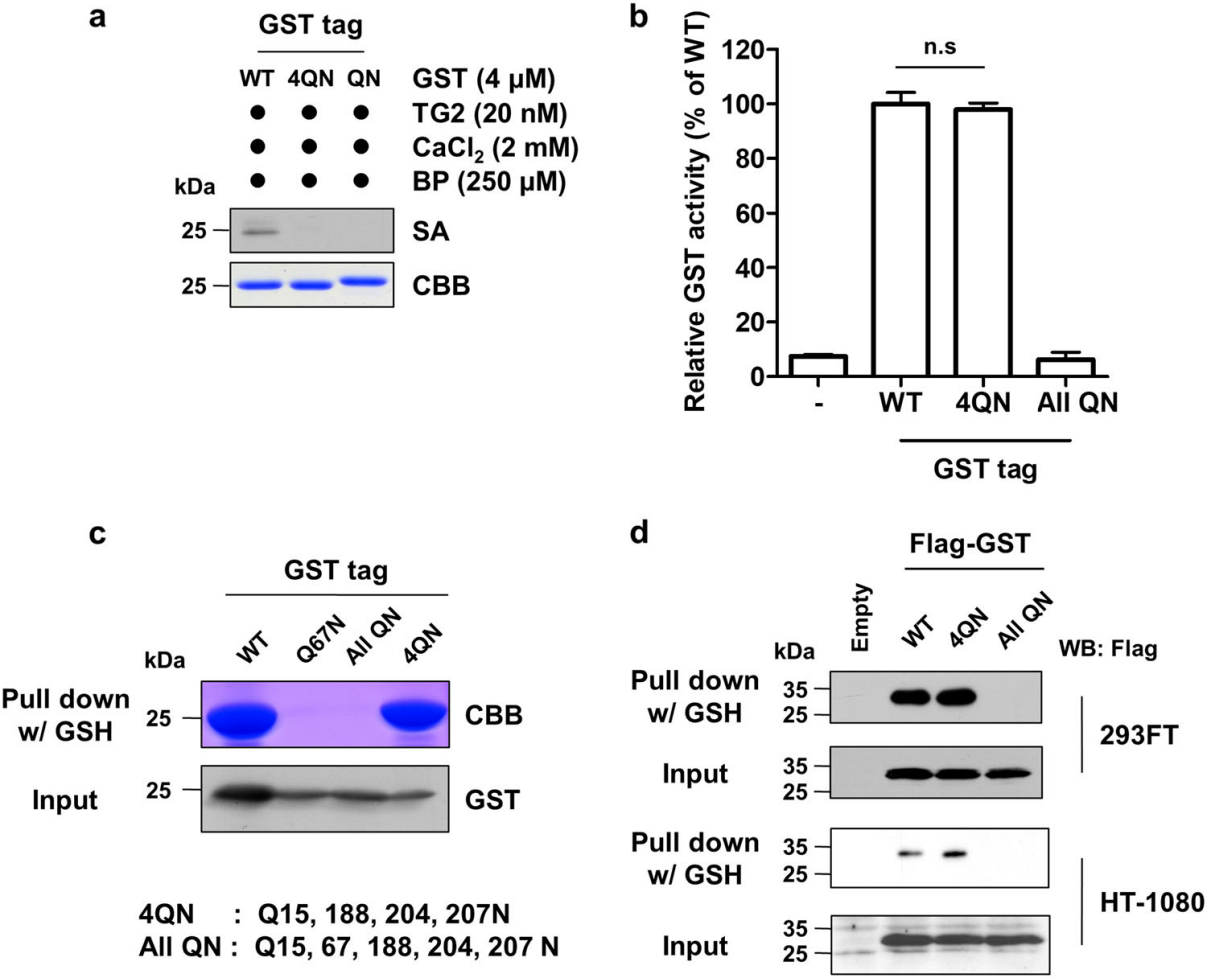
A polyamination-defective GST tag was purified by GSH affinity chromatography. **a** Generation of a polyamination-defective GST tag. Four polyamination sites and all glutamine residues in the GST tag were mutated to asparagine (4QN: Q15, 188, 204, 207 N, All QN: Q15, 67, 188, 204, 207 N). The GST tag was then cloned into a His-tag vector, and the His6-WT and His6-mutant GST tags were expressed in *E. coli*. The tags were purified with Ni-NTA resin and incubated with human TG2 (20 nM), BP (250 μM), and CaCl_2_ (2 mM) at 37 °C for 1 h. Protein in the reaction mixtures was then separated by SDS-PAGE and analyzed by Coomassie Brilliant Blue (CBB) staining or western blotting with SA-HRP. **b** The GSH binding affinity of GST^4QN^ and GST^allQN^ was evaluated by measuring GST enzymatic activity using a CDNB assay. **c** The Q^67^ residue in the GST tag is required for GSH affinity purification. Wild-type and mutant GST tags were purified with GSH-Sepharose beads. GST tags were separated by SDS-PAGE and quantitated by CBB staining or western blotting with an anti-GST antibody. **d** Purification of the GST tag that was expressed in various cell lines. Lysates were prepared from 293FT and HT-1080 cells that were transfected with the pCMV-2B expression vector (FLAG-tag). Prepared lysates were mixed with GSH-Sepharose beads. Purified GST-tagged proteins were analyzed by western blotting with an anti-FLAG antibody.

It is important to determine whether polyamination-defective GST (GST^4QN^) can be purified by affinity chromatography before it can be used as a tag. Thus, we generated two mutants, GST^4QN^ (Q15, 188, 204, 207 N) and GST^allQN^ (Q15, 67, 188, 204, 207 N), by site-directed mutagenesis. The mutants were cloned into a His6-tagging vector. His6-WT and His6-mutant GST tag were purified with Ni-NTA resin. Purified GST tag proteins were incubated with TG2 and BP. Western blotting with SA-HRP demonstrated that GST^4QN^ and GST^allQN^ were not polyaminated by TG2 (Fig. 4a). Next, we measured GST enzymatic activity to assess the effect of a Q-to-N mutation in GST on GSH binding affinity and protein folding. GST^WT^ and GST^4QN^ exhibited similar enzymatic activity, and GST^allQN^ activity was not detectable (Fig. 4b). These data indicated that mutating polyamination sites had no effect on GSH binding affinity. The difference between GST^4QN^ and GST^allQN^ is the Q67 residue, and as noted in our earlier results (Fig. 1c), GST^Q67N^ could not be purified with GSH-Sepharose beads. This finding suggests that the Q67 residue is critical for affinity purification. To confirm these results, we compared the ability of GST^WT^, GST^Q67N^, GST^4QN^, and GST^allQN^ to bind to GSH-Sepharose beads. CBB staining and western blotting with an anti-GST antibody revealed that both the GST^WT^ and GST^4QN^ proteins were pulled down by GSH-Sepharose beads, but GST^Q67N^ and GST^allQN^ were not. There was no significant difference in purification yields between the GST^WT^ and GST^4QN^ proteins (Fig. 4c). Therefore, the GST^4QN^ mutant is a polyamination-defective GST that could be used for affinity purification with GSH-Sepharose beads.

Next, we compared the purification yield of the GST^WT^ and GST^4QN^ proteins. HT-1080 (high TG2 expression) and HEK293FT (low TG2 expression) cells were transfected with FLAG-tagged GST^WT^, GST^4QN^, and GST^allQN^ constructs. The FLAG-GSTs were purified by pulldown with GSH-Sepharose beads. Western blot analysis showed higher levels of purified GST^4QN^ protein than the levels of purified wild-type GST in HT-1080 cells, which express higher levels of TG2, than HEK293FT cells (Fig. 4d). These results demonstrated that GST^4QN^ could be used to purify GST fusion proteins regardless of the TG2 expression levels.

### GST^4QN^ increases the protein–protein interaction of fused p53 in cells

**Fig. 5:**
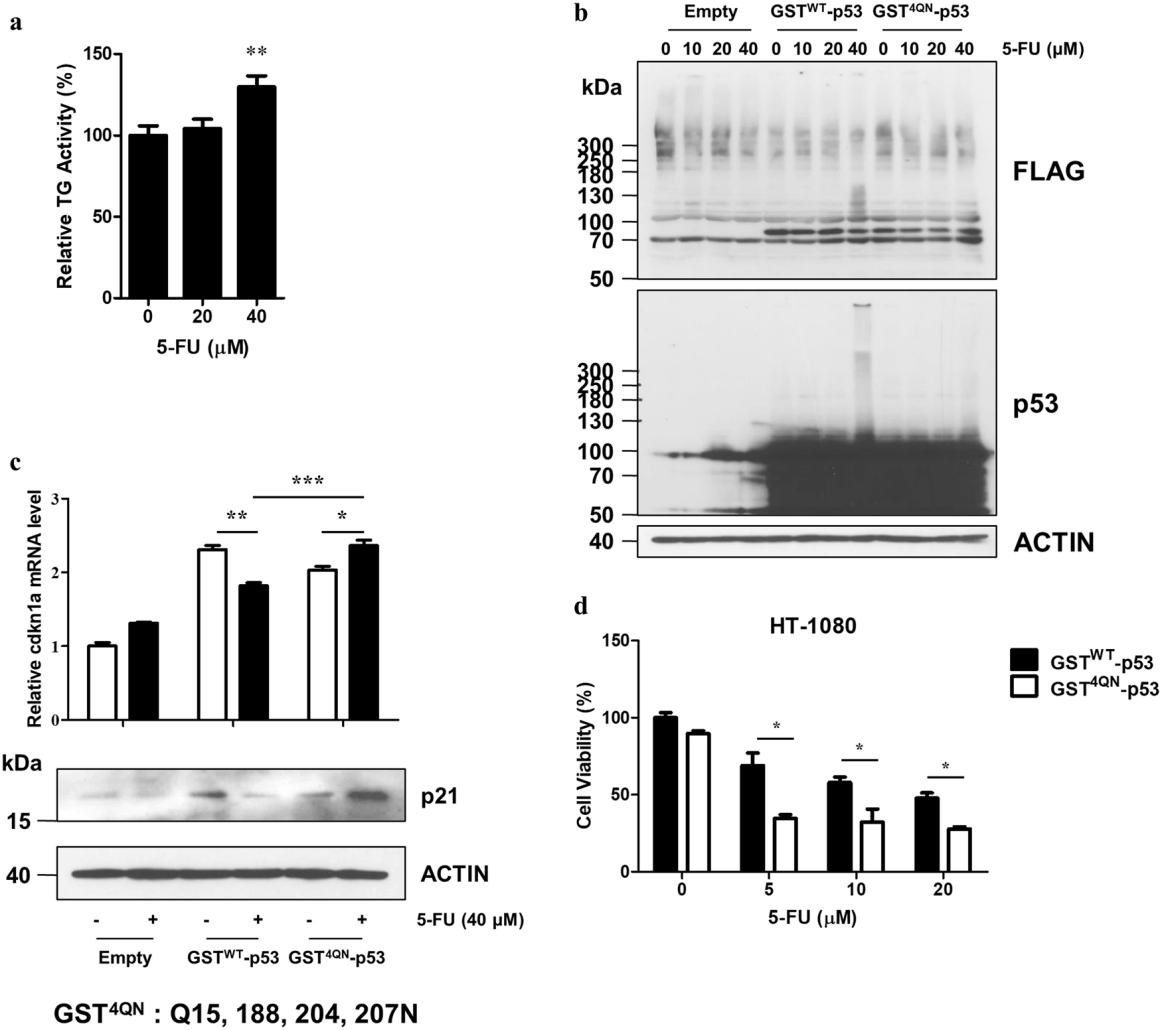
GST^4QN^ maintains fused p53 function in cells. **a** TG2 activity levels in H1299 cells treated for 6 h with 5-fluorouracil (5-FU) were measured with a well plate assay. **b** Decreased crosslinking of GST^4QN^-fused p53. H1299 cells were transfected with the pCMV-2B vector, which encodes p53 that is fused with either GST^WT^ or GST^4QN^. The cells were then treated with 5-FU for 6 h. Cell lysates were prepared and analyzed by western blotting with anti-FLAG, p53, and β-ACTIN antibodies. **c** The p21 mRNA and protein levels in H1299 cells that expressed p53 fused with either GST^WT^ or GST^4QN^ were assessed by RT-qPCR or western blot analysis, respectively. β-ACTIN was used as an internal control. The 5-FU treatment duration was 12 h. **d** HT-1080 cell viability after transfection with either GST^WT^-p53 or GST^4QN^-p53 and treatment with 5-FU for 24 h was evaluated with a WST-1 assay. Statistical significance is represented in the figures by *p < 0.05; **p < 0.01; ***p < 0.001.

GST fusion proteins are used to identify novel protein interactions. First, we examined the interaction between the GST tag and human cellular GST isoforms. When the molecules pulled down using the Flag-tagged GST tag were analyzed, no interaction was detected between the GST tag and cellular GSTs (Supplementary Fig. S3). As shown in Fig. 2a, TG2 induces polymer formation of the GST tag. We tested whether TG2-mediated GST crosslinking affected the ability of the fused protein to interact with other proteins. FLAG-GST^WT^-p53 and FLAG-GST^4QN^-p53 expression constructs were generated and transfected into p53-deficient H1299 cells. Next, p53 function was compared after treatment with 5-fluorouracil (5-FU). Because TG2 is activated by DNA damaging agents^16^, we measured TG2 activity in H1299 cells that were treated with 5-FU and found that TG2 activity increased when the concentration of 5-FU was >40 μM (Fig. 5a). Western blotting with anti-FLAG and anti-p53 antibodies revealed that 5-FU treatment induced the polymer formation of GST^WT^-p53 but had no effect on GST^4QN^-p53 in H1299 and HT-1080 cells (Fig. 5b and Supplementary S4). Interestingly, in HT-1080 cells, which show higher TG2 activity than H1299 cells (Fig. 3a), a lower dose of 5-FU was required to induce the formation of multimers, suggesting that the formation of GST tag aggregates can be determined based on the degree of cellular TG2 enzyme activity. When p53 transcriptional activity in response to 5-FU treatment was measured, the mRNA and protein levels of p21, which is a p53 target gene, were decreased in the GST^WT^-p53-expressing cells. Notably, p21 levels were increased in the GST^4QN^-p53-expressing cells compared to the untreated cells (Fig. 5c). Moreover, 5-FU treatment decreased the viability of the GST^4QN^-p53-expressing cells compared to the GST^WT^-p53-expressing cells (Fig. 5d). Our results indicated that TG2 interferes with GST fusion protein interactions. Namely, protein–protein and protein-DNA interactions are disrupted by GST tag crosslinking, which results in functional inactivation of the fused protein. Thus, a GST^4QN^ fusion tag is recommended to prevent aberrant pulldown experiments.

## Discussion

Identification of protein interactions is a strategy used to predict the functions of an unknown protein, to explore a novel role for a known protein in cellular processes and to identify a new member of protein complexes^17^. In this approach, a recombinant protein of interest is commonly fused to a variety of tags (including the His6-tag, maltose-binding protein, and GST) to detect, purify, and pull down the interacting partners of a protein of interest. Fusion tags are also useful to increase production yield because they enhance the solubility and proper folding of recombinant proteins that are expressed in *E. coli*^18^. It has been observed, however, that fusion tags that are expressed in cells are cleaved by cellular proteases, which suggests that these tags can be modified by endogenous enzymes during experimental procedures. Tag modification could potentially interfere with protein–protein interactions and subsequent pulldown assays^19^.

GST from *S. japonicum* is one of the most widely used tags. This tag is advantageous because of single-step affinity purification methods and because purification of the GST fusion protein can be easily monitored by measuring enzymatic activity. Thus, purified GST fusion proteins are commonly used to study protein–protein interactions. Despite its advantages, GST is a dimeric enzyme, and its crystal structure reveals four exposed cysteine residues that can be easily oxidized to form aggregates^20^. These features limit the use of GST for oligomeric protein tagging and for studying protein–protein interactions under oxidative stress conditions. We also observed increased aggregate formation following incubation of the GST fusion proteins with cell lysate, which appears to depend on both the cell type and experimental conditions. However, it is unclear why the aggregation of GST fusion proteins increases under specific circumstances.

Our results demonstrated that TG2 mediates GST tag crosslinking or polyamination and causes aggregate formation. Furthermore, GST fusion proteins are susceptible to TG2-mediated aggregation, independent of the specific fused protein that is used. Interestingly, TG2-mediated GST polyamination had no effect on its GSH binding affinity or its GST enzymatic activity. With site-directed mutagenesis, four Q residues in the GST tag were identified as polyamination sites. Replacement of Q residues with N residues at the polyamination sites had no impact on GSH binding affinity or GST enzymatic activity compared to that of wild-type GST. Polyamination sites are not conserved in the GST family, and proteins that contain a TG-like domain are not found in the *S. japonicum* genome. These findings suggest that a GST tag is not an authentic TG2 substrate but instead is accidentally crosslinked by TG2 during cell lysate incubation. Indeed, because more than 100 proteins have been reported as TG2 substrates and because TG2 has broad substrate specificity, the GST tag could be crosslinked with a number of cellular proteins^21^. In addition, the N-terminal thioredoxin-like domain of GST, known as the G-site, is responsible for GSH binding^22^. At the G-site, tyrosine (alpha, mu, pi, and sigma isoenzymes), serine (kappa), or cysteinyl residues (theta) are involved in GSH binding through hydrogen bonding. In contrast, our results demonstrated that the Q^67^ residue is critical for GSH binding. This finding suggests that an interaction between tyrosine (Y^60^) and the Q^67^ residue of GST may contribute to GSH binding.

The cellular availability of Cys and bidirectional flux of cellular thiols across the plasmalemma may influence the interaction of the GST tag. Under oxidative stress conditions, glutathione disulfide (GSSG) and GSH conjugates are exported into the extracellular space for redox homeostasis and detoxification^23^. Because TG2 activity is highly associated with cellular redox status and alterations in the bidirectional flux of cellular thiol modulate TG2 activity, regulation of thiol flux in cells probably affects the TG2-mediated aggregation of the GST tag and its interaction with cellular proteins. Therefore, the cellular redox status needs to be considered for pulldown experiments using GST fusion proteins to detect protein–protein interactions.

Cellular human GST monomers physically interact with various cellular proteins. GST plays roles in signal transduction pathways via interaction with signaling kinases such as JNK and Cdk2; regulation of cellular redox status via interaction with peroxiredoxin 6; and regulation of gene expression via interaction with transcription factors such as STAT3 and Nrf2^24^. Among cellular GSTs, GSTP1 was reported to form a complex with TG2^25^ and be multimerized by TG2^26^. Therefore, it is probable that TG2-mediated modification of GSTP1 affects the interactome of cellular GSTP1, resulting in an alteration of interactions with its binding partners. The exact molecular mechanisms and functional consequences of TG2-mediated modification of cellular GSTP1 need to be further investigated.

In summary, our results demonstrated that TG2 in cell lysates causes GST aggregate formation through crosslinking activity, which interferes with pulldown experiments and alters fused protein function. We conclude that a GST^4QN^ tag could improve the reproducibility and reliability of protein–protein interaction studies without compromising GSH affinity.

## Supplementary information

Supplemental Materials
