# Supplementary material for: Transglutaminase 2 crosslinks the glutathione S-transferase tag, impeding protein–protein interactions of the fused protein
Source: Exp Mol Med. 2021 Jan 13;53(1):115–24. doi: 10.1038/s12276-020-00549-9 (PMC8080825; doi:10.1038/s12276-020-00549-9)
Supplement: Supplementary file 1 — Supplemental Materials [file 12276_2020_549_MOESM1_ESM.pdf]

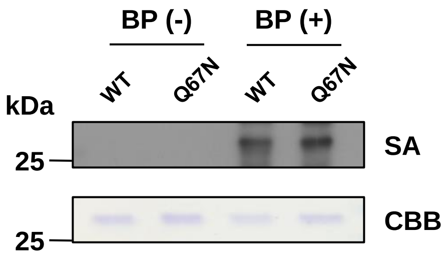

Supplementary Figure S1. GST Q67 residue is not modified by transglutminase 2.

In vitro TG2-mediated BP incorporation assay. His-tagged GST Wild-type and Q67N mutant (4 μM) were incubated with purified human TG2 (50 nM) in the presence or absence of BP (500 μM) at 37 °C for 1 hour. The reaction mixtures were analyzed by Coomassie Brilliant Blue (CBB) staining and western blotting with streptavidin (SA)-HRP.

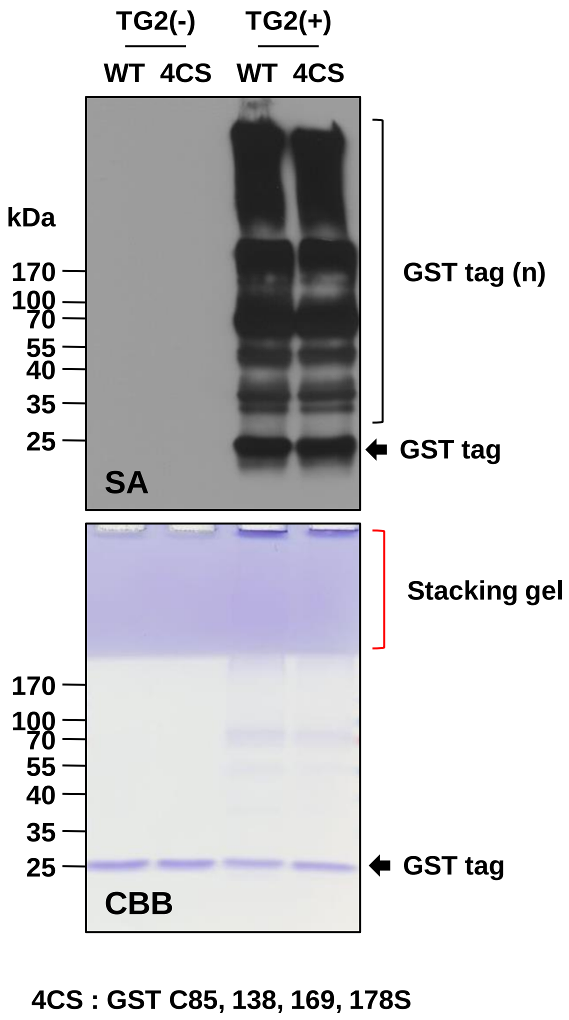

Supplementary Figure S2. GST cysteine residues are not required to TG-mediated GST modification.

TG2-mediated crosslinking of GST wild-type and 4CS (C85, 138, 169, 178S) mutant. GST wild-type and 4CS mutant (5 µg) were incubated with purified human TG2 (2 µM) in the presence or absence of BP (500 µM) at 37 °C for 1 hour. The reaction mixtures were quantified by Coomassie Brilliant Blue (CBB) staining and western blotting with streptavidin (SA)-HRP.

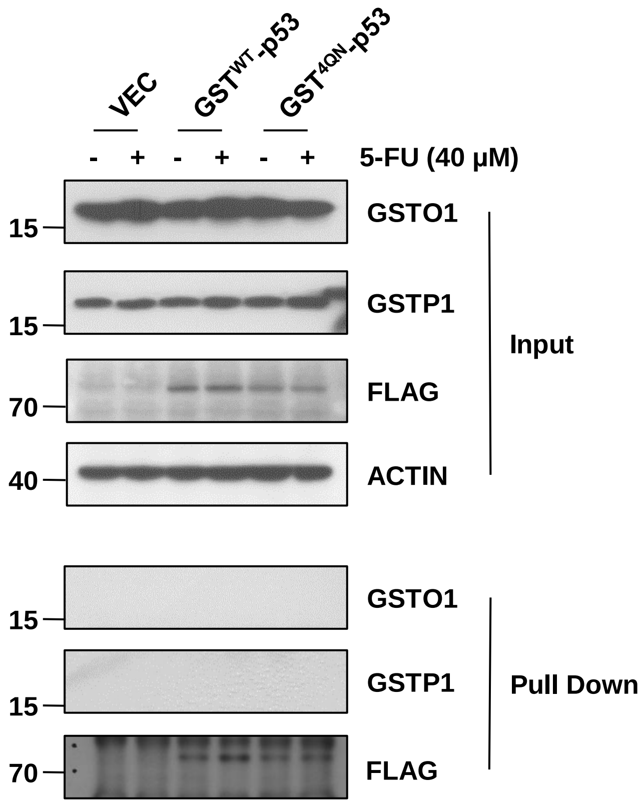

Supplementary Figure S3. Endogenous GSTs do not interact with GST-tagged p53.

H1299 cells were transfected with the pCMV-2B vector, which encodes for p53 that is fused with either wild-type or GST^{4QN}. The cells were then treated with 5-FU for 12 hours. Wild-type and mutant GST-p53 transfected lysates were incubated with FLAG beads for overnight at 4 °C. Prepared samples were analyzed by western blotting with anti-FLAG, GSTP1, GSTO1 and anti-ACTIN antibodies.

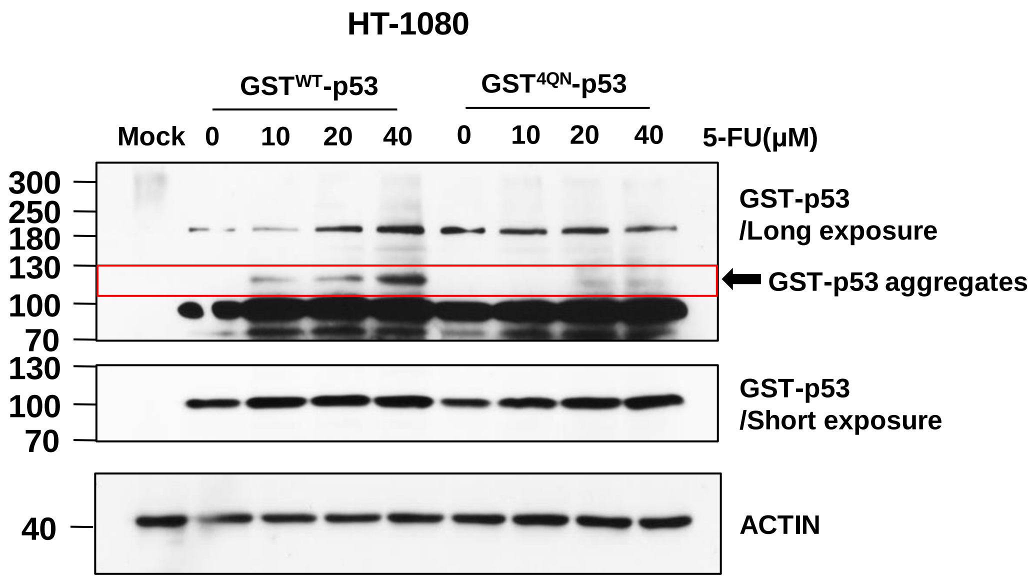

Supplementary Figure S4. Cross-linking of GST^{WT} but not GST^{4QN} in HT-1080 cells.

TG2-mediated crosslinking of GST^{WT}-p53 in HT-1080 cells. HT-1080 cells were transfected with the pCMV-2B vector, which encodes for p53 that is fused with either wild-type or GST^{4QN}. The cells were then treated with 5-FU for 24 hours. Cell lysates were prepared and analyzed by western blotting with anti-ACTIN and anti-p53 antibodies.
